# Multisensory integration and belief in the self

**DOI:** 10.3389/fpsyg.2022.983592

**Published:** 2022-10-13

**Authors:** Rafael Bretas, Banty Tia, Yumiko Yamazaki, Atsushi Iriki

**Affiliations:** Laboratory for Symbolic Cognitive Development, RIKEN Center for Biosystems Dynamics Research, Kobe, Japan

**Keywords:** somatosensory integration, mirror self recognition, consciousness, credition, beliefs, parietal cortex, self recognition

## Introduction

Our first experience of the world originates from the information we receive through the senses, allowing us to make mental representations of the features that can be experienced from each part of the environment—be those objects, events, places, or beings. However, these parts are not perceived separately through each sense. Rather, sight, touch, smell, hearing, and taste are integrated early in life in multimodal areas in the brain (Lewis and Essen, 2000). While this process, together with memory, supports the formation of beliefs of increasing complexity, it is also constantly modified by those same beliefs. In this opinion paper we briefly describe some of the neural underpinnings of conscious perception and illustrate how a complex belief is formed from sensory information using the example of mirror self-recognition in macaques.

From when a sense is raised to awareness until when it is integrated into other senses, a separate process occurs. A relational association is established, one in which the codependency of these stimuli becomes their own defining characteristic. That is, an object or event is recognized by simultaneously eliciting different modalities of sensation (Crick and Koch, 1990; Deroy et al., 2016). It is important to clarify that multisensory integration does not necessarily induce a conscious process. However, unconscious integration seems to be possible in only limited conditions, such as in simple forms of visual adaptation or when a stimuli pair has been previously learned (Faivre et al., 2014; Mudrik et al., 2014).

In this sense, integration can be understood as an antecedent to a behavior, perhaps similarly to how attitudes or mindsets are modulated (Seitz and Angel, 2012) or as an *empirical belief* (Seitz and Angel, 2020). Nevertheless, becoming aware of a percept as an amalgam of sensations forms the basis for a conscious belief that can be expressed as a decision or action, or in declarative form as a *conceptual belief* (Seitz and Angel, 2020). From the point in which a sense is raised to awareness, recognition may take place. Although often understood as a single behavioral phenomenon, “recognition” arises from separate neurophysiological processes that can function independently of each other. Here, we will focus on two of these general processes that are often taken as determinators of recognition.

First, there is a memory component that locates the sensory information in the place and context where sensation occurs (Mandler, 1980), supported by connections between sensory areas in the neocortex, perirhinal cortex, and parahippocampal regions (Brown and Aggleton, 2001; Eichenbaum et al., 2007). This system, or systems, as it could be subdivided in two main components, is responsible for retrieval of contextual information and the recollection of the stimulus (Brown and Aggleton, 2001). Autobiographical memory also centers sensory experiences around oneself as the individual agent of sensation, perhaps mediated by connections between the posterior cingulate and medial parietal cortex (Rolls, 2022). The hippocampal and parahippocampal regions show extensive connections to sensory and motor areas, but despite playing a fundamental role in recollecting and situating the sensory information received in time and space, recognition itself seems to be formed independently from these regions. Moreover, lesion experiments confirm that memory is not necessary for simple object recognition (DeCoteau and Kesner, 1998; Burwell, 2000; Save and Poucet, 2000; Langston and Wood, 2010), instead it may represent the emotional contents and semantic information rather than the physical properties that allow the conscious perception of an object (Rolls, 2022; Rolls et al., 2022).

The second function that supports recognition is multisensory integration. The perception of simple physical features, such as shape, color, or texture, can be accomplished by unimodal tactile and visual processing streams without reaching awareness. Unconscious perception is also common in multimodal areas for the purposes of guiding motor control (Milner and Goodale, 2008; Mudrik et al., 2014). However, recognition (i.e., the conscious perceptual experience that allows the identification of an object or scene) recruits large, distributed networks that integrate different senses (Dijkerman and de Haan, 2007; Winters and Reid, 2010). The parietal cortex appears to be the source of this conscious perception process, being well interconnected with prefrontal, cingulate and primary sensory areas (Lewis and Essen, 2000; Vincent et al., 2006; Whitlock et al., 2008; Rolls et al., 2022). Furthermore, the parietal cortex, together with the prefrontal cortex, directs attention and modulates perception and the emotional significance of sensory events (Mesulam, 1998; Steinmetz et al., 2000; Culham and Kanwisher, 2001; Galletti et al., 2010). In humans, the parietal cortex is also functionally interconnected with language and declarative memory areas, a hallmark of conscious perception (Rolls et al., 2022).

This wide network that combines perception and memory to contextualize what is perceived may form a part of the broader consciousness, allowing one to recognize the world and the self (Dehaene and Changeux, 2011; Mudrik et al., 2014). The large-scale synchronization between sensory, motor and executive functions required for conscious perception could be mediated by the claustrum, as an area with reciprocal connections to most regions of the cortex, perhaps as a conductor of sensory experiences (Crick and Koch, 2005), with this combination process being permeated by different degrees of beliefs (Seitz and Angel, 2012).

Here, we would like to focus on the formation and modulation of some simple beliefs related to the self and the world. For example, when there is a mismatch between the senses, such as in the ventriloquist effect, localizing the source of a sound together with a movement (mouth movement, in this example) uses a combination of both auditory and visual senses, but viewers' sensations are distorted by beliefs of how reality *should* be according to previous experiences (Alais and Burr, 2004; Seitz and Angel, 2020). The viewer knows that the sound comes from the ventriloquist, not the puppet, but at the same time they also have experienced a reality in which sounds usually come from moving parts, and in particular, voices come from moving mouths.

This type of sensory conflict is clearer in the rubber hand illusion. Even when there is a stable, conscious belief that the hand seen is not one's own, a false belief associated with a recent sensation may override the belief. The effect is strong enough to cause the activation of somatosensory areas in the brain when the false hand is stroked (Ehrsson et al., 2005). Such cases could be an example of how a conscious declarative belief can be distorted by a sensory belief. In both cases the causes could maybe be reduced to the single, deeply rooted, unconscious belief intrinsic to the senses involved that sight has higher spatial accuracy than sound or touch. Therefore, sight *should* be more reliable when incongruent spatial judgements are involved.

There are also cases where sensation is not inconsistent but instead ambiguous, such as in multistable perception (i.e., when two concurrent percepts spontaneously change). In these situations, prior beliefs may act in harmony with newly acquired sensory information to guide attention and modulate perception (Sterzer et al., 2009; Conrad et al., 2012), with the speed and often mutually exclusiveness of these changes being noteworthy.

## The self in the mirror

Self-recognition in the mirror was proposed by Gallup in 1977 as “a technique for providing empirical and operational substance to the existence of self-awareness” (Gallup, 1977). Despite debates over how much self-awareness intersects with mirror self-recognition, the mirror offers the perfect example of the development of different categories of belief and how they interfere with each other according to the criteria of Seitz and Angel (2020). Since humans appear to develop or be guided into mirror self-recognition very early in life, it may be difficult to imagine its emergence. Macaques however, although not having innate self-recognition, can acquire it through training and habituation (Chang et al., 2017; Bretas et al., 2021). Therefore, below we follow the trajectory of a hypothetical macaque acquiring such a skill based on our own experimental observations (Bretas et al., 2021).

**Figure 1:**
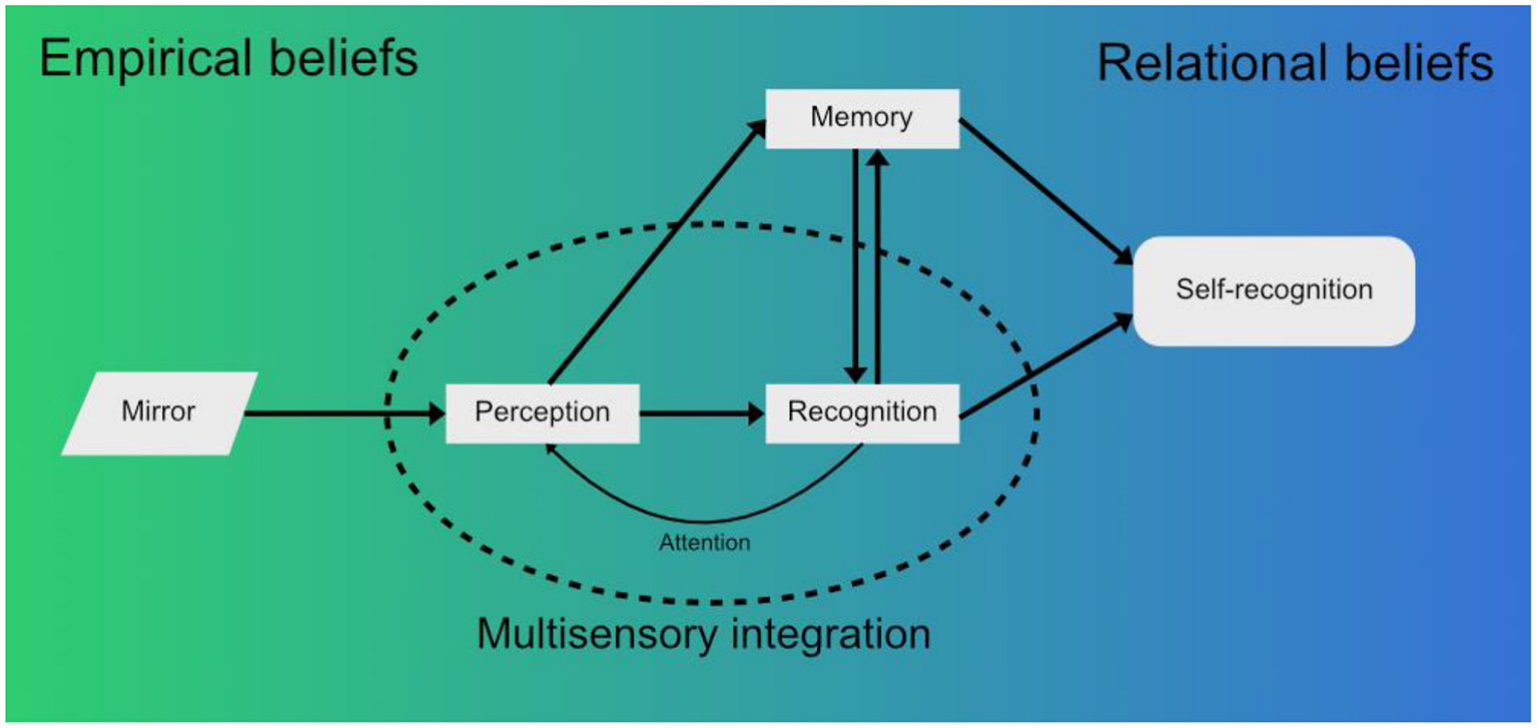
When a macaque looks into a mirror, perception of the raw visual information from the mirror forms a first approximation of reality in the form of *empirical beliefs* (Seitz and Angel, 2020). This visual stimulus is integrated with other sensory modalities, such as the proprioception of the macaque's own limbs moving, and raised to conscious awareness. Recognition of the mirror image then takes place - initially as another macaque, not the self. Simultaneously, the novelty, emotional significance and other aspects associated with the stimuli are recollected from memory—previous experiences with other macaques, the novelty of the individual seen in the mirror, innate fear, etc. The associations between what is perceived and the environment form *relational beliefs* (Seitz and Angel, 2020). These beliefs are being updated constantly according to new sensory information received from the environment, which also feedbacks into perception through attention mechanisms. Finally, a complex belief that matches the perceived stimuli may evolve, the belief that the macaque in the mirror is a reflection of the self. This belief is expressed in the form of mirror self-recognition behaviors (Chang et al., 2017; Bretas et al., 2021), but in humans it could further develop into *conceptual beliefs*, discrete, language bound concepts (Seitz and Angel, 2020) (e.g.: “I am the person in the mirror and I appear to others are they appear to me”).

At the lowest level, there is the visual stimulus imparted by the mirror as the sensory reality (i.e., raw information that contains the visual features of the subject in the mirror). Innate visual mechanisms combined with the memory mechanisms described before allow for the classification of this stimulus (*The image in the mirror moves. It's a live being. It is a monkey*.) and its valuation in terms of emotional loading (*Is it a threat? Is it a partner?*). This process can start before the stimulus is raised to conscious awareness and is an example of *empirical beliefs (**Figure 1**)*.

Before self-awareness, one must develop other-awareness, since both processes require the capacity of secondary representation (Asendorpf and Baudonnière, 1993). Both other beings and the self-body are directly accessible through the senses, but even accounting for physical similarities, there is a significant difference in perspective. At the most basic level, the spatial organization of the body will appear to be different, with one not being able to see their own face or back, for example. There is also a difference in the integration of the senses: visually the self and the other can be equally perceived, but touch is limited to the self. For example, while two people can see each other touching an object, the touch sensation can only be felt by the agent. In the same way that self-body awareness arises from the association between different unimodal sensations, such as vision and touch (Apps and Tsakiris, 2014), mirror-self recognition relies on these same sensory mechanisms as precursors.

When the macaque looks into the mirror, a third-person view of the self is shown. This view elicits new beliefs, both conflicting and ambiguous, as described in the previous section. While the body in the mirror appears from the same perspective as that of others, its movements can be precisely controlled, which is a sense of agency over a distant subject. The same could be said about the touch sensation, which now can be felt by both the agent and the mirror-image. The subject now may recollect that the primate in the mirror is always the same subject with the same face. It also does not feel like the primate's previous experiences or what is expected of the related visual stimuli. The mirror shows soft fur, but it is cold and hard to the touch; the primate in the mirror cannot be touched. These new sensorial experiences accumulate and generate *relational beliefs* about the relations with the environment. According to Sugiura et al. (2015), mental representations are formed through the association between an action and its consequential perception learned through repeated experience.

Multimodal visuo-somatosensory neurons are often spatially tunned to represent the space around the subject from an egocentric point of view, mapping the position of the own body and reachable objects nearby. These neurons estimate and guide limb movement as well as tracking objects moving toward or nearby the subject (Taoka et al., 2013; Hihara et al., 2015; Galletti et al., 2022). But when movement is performed in front of the mirror, the mismatch between the prediction and the actual sensation may no longer appropriately represent the actual bodily or environmental state (Sugiura et al., 2015). Perception may then be updated by this new incoming sensory data and combined with past outputs and decisions to account for this new state, in accordance with the free-energy principle (Apps and Tsakiris, 2014); *relational beliefs* can alter *empirical beliefs*. This update to sensation may lead to the mirror being ignored from that point on as a useless social cue, with no new attempts to interact with this primate in the mirror and no emotional reactions of fear or dominance upon seeing it. However, discrepancy from previous beliefs in this new information received from unimodal areas could be explained away by multisensory integration, giving rise to self-recognition (Apps and Tsakiris, 2014; Chang et al., 2017; Bretas et al., 2021). Indeed, binding different sensory aspects of and object in a mutually coherent way provides the experience of perceptual unity necessary to group the individual body parts in a concept of an indivisible self-body (Crick and Koch, 1990; Bretas et al., 2021).

The acknowledgment of the self in third-person promotes the belief that the other is like the self, with empathy and the emotional valence of the new beliefs further shaping both mirror-perception and own-perception (Gallup, 1998; Sugiura et al., 2015; Bretas et al., 2020). Primates are special in that their brains evolved with the addition of new functional subdivisions to the neocortex (Dooley and Krubitzer, 2013). Areas in the parietal cortex related to self-awareness and social-awareness may be essential to the development of mental models of introspectively based social strategies and language, forming the basis for *conceptual beliefs* and culture (Gallup, 1998; Sugiura et al., 2015; Bretas et al., 2020, 2021; Seitz and Angel, 2020; Seitz, 2022). *Conceptual beliefs*, thereupon, give support to meta-beliefs, elevating relational beliefs about the other to language-bound discrete concepts (e.g., “the other believes…”) to achieve a comprehensive notion of the world grounded in internal representations of the physical, social, and cultural environments (Angel and Seitz, 2016; Bretas et al., 2020; Seitz and Angel, 2020).

## Author contributions

RB and AI contributed to conception and theory of the study. RB wrote the first draft of the manuscript. BT, YY, and AI wrote sections of the manuscript. All authors contributed to manuscript revision, read, and approved the submitted version.

## Funding

The authors declare that this study received funding from Dr. Rüdiger Seitz, via the Volkswagen Foundation, Siemens Healthineers, and the Betz Foundation. The funders were not involved in the study design, collection, analysis, interpretation of data, the writing of this article, or the decision to submit it for publication.

## Conflict of interest

The authors declare that the research was conducted in the absence of any commercial or financial relationships that could be construed as a potential conflict of interest.

## Publisher's note

All claims expressed in this article are solely those of the authors and do not necessarily represent those of their affiliated organizations, or those of the publisher, the editors and the reviewers. Any product that may be evaluated in this article, or claim that may be made by its manufacturer, is not guaranteed or endorsed by the publisher.
